# Cannabis tourism in transition: operators’ perspectives on regulatory change and governance in Bangkok, Thailand

**DOI:** 10.1186/s42238-026-00462-4

**Published:** 2026-06-16

**Authors:** Patreeya Kitcharoen, Shinnawat Saengungsumalee, Pattarachit Choompol Gozzoli

**Affiliations:** 1 Department of Society and Health, Faculty of Social Sciences and Humanities, Mahidol University, Nakorn Prathom, Thailand; 2 Division of Social and Administrative Pharmacy, Faculty of Pharmacy, Siam University, Bangkok, Thailand

**Keywords:** Regulatory change, Cannabis Tourism, Butler’s TALC, Policyscape, Bangkok, Thailand

## Abstract

**Objectives:**

This study examines the perceptions and lived experiences of cannabis business operators in Bangkok as they navigated Thailand’s rapid transition from decriminalization in 2022 to a subsequent medical-only regulatory reversal.

**Methods:**

Using a qualitative design, the researchers conducted semi-structured in-depth interviews with 13 key informants in Bangkok between 2024 and 2025. Participants included retail shop operators (*n* = 8), farm operators (*n* = 3), and policy advocates (*n* = 2). Data were analyzed using the six-phase Thematic Analysis framework supported by ATLAS.ti Version 21.

**Results:**

Five primary themes emerged: (1) Navigating Regulatory Unpredictability and Legal Uncertainty; (2) Structural Inequity and Enforcement Disparities; (3) Market Segmentation and Economic Drivers; (4) Adaptation and Survival Strategies; and (5) Socio-Cultural Perceptions and Public Health. Analysis revealed that while international tourists comprise approximately 60% of the market, domestic customers (40%) are primarily motivated by therapeutic needs. Furthermore, the study synthesized six domains for evidence-based policy, including regulatory formalization, equity promotion, and sustainable cannabis tourism development.

**Conclusions:**

The findings highlight that regulatory instability and the absence of a comprehensive Cannabis and Hemp Act place a disproportionate burden on small Thai operators, creating structural inequities. Additionally, in the absence of formal public health infrastructure, operators have emerged as informal harm reduction actors through proactive customer education and safety guidance. These results offer actionable insights for other jurisdictions navigating similar cannabis policy transitions.

## Introduction

Thailand became the first country in Asia to decriminalize cannabis when, in June 2022, the government formally removed cannabis, kratom, psilocybin mushrooms, and hemp from Category 5 of the Narcotics Act (Narcotics Code). This historic policy shift was preceded by an earlier liberalization: medical cannabis, with no THC restrictions, had been made legal in 2018 under a physician prescription model, and in January 2019 the government allowed cannabis cultivation free from Food and Drug Administration (FDA) oversight (Chambers 2024).

While Thailand’s June 2022 decriminalization framework officially specified that only cannabis extracts containing more than 0.2% tetrahydrocannabinol (THC) by weight remained classified as Category 5 narcotics (Wipatayotin, July 2024), it left raw cannabis flowers completely unregulated, allowing high-potency strains to proliferate in the retail market. Consequently, unextracted plant material of high-potency cannabis strains were legally sold nationwide without potency caps, creating an immediate public health debate regarding product strength and consumer safety (Yimsaard et al. 2023).

The rationale articulated by the Thai government centred on economic recovery following the COVID-19 pandemic, positioning cannabis as a high-value cash crop with the potential to generate up to 1.2 billion US dollars by 2025 (The Straits Times, 2024). Notwithstanding the economic rationale, the decriminalization was affected without a Regulatory Impact Assessment (RIA), which is a standard procedure in Thai legislative reform and is mandated under the Regulatory Impact Assessment guidelines of the Office of the Council of State. The RIA was absence from the 2022 decriminalization meant that the policy was introduced without structured analysis of its economic, social, or public health consequences. Significantly, a Cannabis and Hemp Act would have established a dedicated statutory regime with clear provisions on permissible uses, retail standards, age verification, and enforcement responsibility—distinguishing it from the ad hoc ministerial notifications that governed the sector in practice. With this respect, no comprehensive Cannabis and Hemp Act was enacted for the other substances decriminalised alongside cannabis (kratom, psilocybin mushrooms, hemp) to govern permissible uses, retail standards, or enforcement responsibilities.

Following decriminalization, cannabis retail outlets rapidly proliferated across tourist districts in Bangkok and other popular destinations including Pattaya, Chiang Mai, Samui, Krabi, and Phuket, becoming embedded in a wider supply chain of cannabis products (Clarke & Richmond, 2023; Lertrujwanich et al., 2024). This expansion catalysed the emergence of cannabis tourism—a form of tourism in which visitors engage with cannabis products, services, or experiences in destinations where cannabis use is legally permitted (Osborne and Fogel 2017). Globally, cannabis tourism has gained traction in the Netherlands, Jamaica, Canada, and Uruguay; Thailand’s decriminalization positioned it as a novel destination within this transnational phenomenon. A 2023 report estimated that more than 11 million Thai people had used cannabis products following legalization, of whom approximately 9.9 million reported recreational use compared with 1.1 million for medical purposes (Policy Watch Thai PBS, 2024). Concurrently, the number of mental health patients attributed to cannabis use rose from 306 in 2021 to 934 in 2023, making cannabis the third most common substance associated with mental health presentations after heroin and amphetamines (Policy Watch Thai PBS, 2024). These trends underscore the public health implications of a policy introduced without a clear regulatory framework.

The Thai government’s position subsequently shifted under political pressure. Penalties for recreational cannabis use were introduced, including fines of up to 60,000 baht for consumption and imprisonment of up to one year and/or fines of up to 100,000 baht for commercial sale for recreational purposes (Tepgumpanat & Wongcha-um, 2024). Thailand’s Narcotics Control Board secured a majority vote to reclassify cannabis as a narcotic, and a draft Cannabis and Hemp Bill—intended to restrict use to medical and health purposes only, requiring a physician’s note for access—was anticipated to be passed by Parliament before the end of 2024 (Wipatayotin, 2024). This regulatory reversal created an environment of acute uncertainty for cannabis businesses: operators who had invested in cultivation infrastructure, quality control systems, and retail premises faced an abrupt and unplanned transition from a quasi-liberalized market to a medical-only controlled regime. The policy trajectory has had cascading effects on farmers, communities, industry actors, and international visitors alike.

### Rationale and research gap

Despite the scale and speed of the cannabis tourism phenomenon in Thailand, the lived experiences of cannabis business operators—those most directly affected by the policy transition and its reversal—have received limited scholarly attention. Existing studies have documented the macro-level policy track (Charoenwisedsil et al. 2023; Veilleux 2024) and epidemiological trends in cannabis use (Kalayasiri and Boonthae 2023), but there is a notable gap in qualitative inquiry into how operators have navigated the transition from decriminalization to re-scheduling in real time. Understanding these experiences is essential both for refining the evidence base on cannabis tourism governance and for informing the development of a more equitable and practically implementable regulatory framework in Thailand. Bangkok—as the country’s primary urban tourism hub and the site of the largest concentration of cannabis retail operations—provides an especially relevant setting for such inquiry. This study was conducted during the 2024–2025 fieldwork period, capturing operators’ experiences during the critical transition from a liberalized to a medical-only regulatory regime.

Accordingly, this study sought to: (1) examine the perceptions and lived experiences of cannabis business operators in Bangkok within Thailand’s post-decriminalization regulatory environment, with particular attention to the transition from cannabis legalization to re-scheduling; and (2) develop evidence-based policy recommendations for cannabis tourism governance in Bangkok, grounded in the operational realities and perspectives of industry stakeholders.

### Literature review and theoretical frameworks

#### Cannabis tourism

Cannabis tourism is an emerging and transformative form of tourism facilitated by legalization. Cannabis tourism operations have been documented in multiple regions globally—including Jamaica, the Netherlands, the United States, Canada, Uruguay, and Thailand—became the first country in Asia to enter this space following its 2022 decriminalization. Cannabis tourism occupies a distinctive niche: it is neither a conventionally recognized institutional form of tourism nor a marginal one (Dupej and Nepal 2021; Keul and Eisenhauer 2019). It intersects with existing tourism infrastructure while also generating new forms of social stigma, community concern, and regulatory complexity (Osborne and Fogel 2017). As sovereign states and sub-national regions continue to decriminalized, cannabis tourism has emerged as a distinct market segment. The legal precedents set by early adopters like Canada and the Netherlands provide a comparative baseline for analyzing the evolution of this niche in newly liberalized environments, e.g. Thailand in 2022.

Historically, cannabis in Thailand was classified as a narcotic, with possession or use resulting in criminal penalties, and its discourse was shaped by the gateway drug theory (Sirita 2022). Against this backdrop, the abrupt shift to decriminalization created conditions in which recreational cannabis businesses proliferated rapidly in tourist districts—most notably in Bangkok’s Silom, Sukhumvit, and Khao San Road areas—before the subsequent regulatory clampdown. From a health perspective, cannabis’s active compounds, including tetrahydrocannabinol (THC) and cannabidiol (CBD), have established therapeutic applications in alleviating pain, managing anxiety and depression, and treating conditions such as glaucoma and insomnia under medical supervision (Osborne and Fogel 2017). From an economic perspective, legalization creates tax revenue and market opportunities comparable to those of alcohol and tobacco—opportunities that Thailand’s decriminalization briefly, if incompletely, realized.

Following legalization, the cannabis sector experienced rapid expansion across the entire supply chain, with recreational products driving the majority of this growth (Kanato et al. 2020; Sommano et al. 2022; Zinboonyahgoon et al. 2021). Cannabis dispensaries quickly proliferated throughout every corner of key commercial zones. This boom catalyzed adjacent markets, leading to a diverse range of cannabis-infused edibles, such as candies, sweets, foods, and beverages. Concurrently, the apparel industry began utilizing hemp fibers to create unique fashion lines and textiles. Cannabis also integrated deeply into the tourism and hospitality sectors through the emergence of specialized wellness services, including cannabinoid therapies, themed spas and massages, and traditional medical clinics. However, this rapid commercialization occurred without sufficient product regulation, triggering widespread public health concerns at a national level. Stakeholders have expressed particular anxiety regarding the potential risks posed to the youth population, even though a direct link proving cannabis functions as a gateway to harder drugs remains unsubstantiated.

Unfortunately, the new legislation was not complemented by a public health perspective (Yimsaard et al. 2023). High-potency cannabis is directly linked to higher rates of usage frequency, chemical dependence and the onset of psychiatric disorders in young people. Thus, future cannabis regulation in Thailand must prioritize the monitoring of product potency and its specific health impacts on the youth population to be effective.

#### Theoretical framework I: Butler’s Tourism Area Life Cycle (TALC)

The development of cannabis tourism in Bangkok is analyzed through the lens of Butler’s (Butler 1980; 2025) Tourism Area Life Cycle (TALC) model. This model describes how tourist destinations pass through a sequence of stages—exploration, involvement, development, consolidation, and stagnation—each presenting distinct policy challenges and opportunities. The destination life cycle may be understood as a sequential process comprising several interrelated stages. In the exploration stage, tourism activity remains limited in scale, with only a small number of visitors and minimal infrastructure, often relying on basic or easily developed facilities. This is followed by the involvement stage, during which local communities begin to participate more actively in tourism by providing basic services and amenities for visitors. As tourism demand expands, the destination enters the development stage, characterized by rapid growth, increasing external investment, and a substantial rise in visitor numbers, which may eventually exceed the size of the local population. The consolidation stage occurs when the destination becomes well established and the rate of growth begins to slow or stabilize. Finally, the stagnation stage is reached when visitor numbers peak or begin to decline, often as the destination’s carrying capacity becomes evident and its attractiveness diminishes over time. Traditionally, destinations progress through stages from exploration to stagnation based on carrying capacity and market saturation. However, the Thai case presents a unique “policy-driven life cycle” (Veilleux 2024).

Following the 2022 decriminalization, cannabis tourism has developed on its own and give significant impacts on Thailand’s tourist destinations (Phucharoen et al. 2023). Considerably, Bangkok bypassed the gradual ‘involvement’ phase, moving directly into a rapid development stage characterized by high-density retail clustering in Sukhumvit, Kao san, and Silom. The recent government turns toward re-scheduling cannabis as a narcotic represents a premature, state-mandated stagnation phase. The trajectory of Bangkok’s cannabis sector aligns with recent critiques of the TALC model, which suggest that institutional interventions and the policy-driven disruptions can abruptly override traditional market-driven cycles (Butler 2025; Veilleux 2024). In the Thai context, this is characterized by a state-induced stagnation, where the ‘cluttered policyscape’ (Mettler 2016) creates a landscape of legal precarity that disrupts the natural progression from the development to the consolidation stage (Hall et al. 2023). While Butler’s (Butler 1980) foundational TALC model offers a robust framework for destination evolution, it exhibits a significant ‘predictive gap’ regarding non-linear, state-imposed disruptions. The model was not designed to accommodate the catastrophic paralysis of global tourism seen during the COVID-19 pandemic, nor the radical regulatory disruption (Veilleux 2024) observed in the Thai cannabis sector. By applying this model to Bangkok, this study explores how politically motivated reversals serve as artificial catalysts for the ‘Stagnation’ phase, independent of traditional market-driven factors.

While the original TALC model did not fully account for magnitude-shifting disruptions like the COVID-19 pandemic or radical politically motivated reversals, it remains a robust tool for examining how institutional interventions shape the adaptive responses of industry stakeholders and the long-term sustainability of the sector. Applied here, Butler’s framework provides a basis for examining how policy intervention at different stages of the cannabis tourism life cycle has shaped operators’ adaptive responses—and what this implies for sustainable development of this emerging sector.

#### Theoretical framework II: The “policyscape” as analytical lens

To account for the multifaceted and often contradictory nature of cannabis governance in Thailand, this study adopts the concept of the “policyscape”. As defined by Mettler (Mettler 2016), a policyscape represents a political environment that is “densely cluttered” with a layered array of historical and contemporary policies. In such landscapes, existing policies often become ineffective or misaligned as socio-political circumstances evolve or issues intensify.

Key to the policyscape framework is the assertion that the efficacy of regulatory interventions is intrinsically tied to local perceptions and individual preferences. From this perspective, policy effectiveness is not a top-down certainty but rather a reflection of how stakeholders adjust their normative judgments to align with specific operational needs (Rustichini and Villeval 2014). The policyscape is conceptualized as a transformative ‘alchemy’ where formal regulations intersect with localized perceptions, socio-economic preferences, and spatial dynamics. In this framework, political and social dimensions represent abstract and practical spheres, respectively; these spaces serve as the arena where diverse policy instruments and stakeholder priorities must simultaneously coexist (Flanagan et al. 2010).

Thailand’s cannabis environment—characterized by overlapping regulatory authorities, competing interests across the public health, agricultural, and economic sectors, and rapid legislative shifts—exemplifies a highly contested and layered policyscape. This theoretical framing allows the study to move beyond viewing policy shifts as isolated events; instead, it situates the lived experiences of operators within a broader, dynamic institutional context of governance.

To capture the dynamic and multifaceted nature of cannabis legalization in Thailand, this study additionally adopts the concept of the “policyscape” (Mettler 2016; Mettler and SoRelle 2018). As noted by Fernandez and Hu (2021) a ‘policyscape’ is characterized by a high density of overlapping and historically layered regulations (Fernandez and Hu 2021).

Therefore, by synthesizing the policyscape with the Tourism Area Life Cycle (TALC), this study can identify how ‘policy-induced’ stagnation occurs not from a lack of market demand, but from the cumulative complexity of a regulatory environment that has become too ‘cluttered’ for local SMEs to effectively navigate. the In the Thai cannabis context, this ‘clutter’ manifests as a conflict between legacy prohibitionist frameworks and modern liberalization efforts. This creates a landscape where policies established at earlier points in time—such as the 1979 Narcotics Act—clash with the rapid-response ministerial decrees of 2022, resulting in the ‘regulatory disruption’ identified by our key informants.

## Methods

### Study design

This study used a qualitative approach. To meet the research objectives of understanding how cannabis business operators perceive their experiences within Thailand’s post-decriminalization regulatory regime and identifying the major themes that characterize their adaptive responses, a qualitative inquiry was selected. Such depth of interpretation is not achievable with quantitative methods. Data were analyzed using Thematic Analysis (TA), as operationalized by Braun and Clarke (2006), a method widely used in health/health policy research for its capacity to generate theoretically rich, substantive findings from qualitative datasets.

### Setting and participants

#### Study setting

Data was collected in Bangkok, Thailand, the heart of the country’s post-decriminalization cannabis industry. The field research ran from 2024 to 2025, a key transition period during which the Thai regulatory environment shifted from a quasi-liberalized, decriminalized regime to a medical-only controlled regime. Against this backdrop, there was a never-before-seen opportunity to document operators’ day-to-day activities in real time.

#### Sampling and participant characteristics

Participants were recruited using purposive sampling, supplemented with snowball sampling techniques. During this policy transition period, purposive sampling ensured the recruitment of informants with immediate experience of how cannabis businesses operated in Bangkok, while snowball sampling helped identify informants who might not be forthcoming publicly about their involvement in the industry. Inclusion criteria were: (a) engagement in Bangkok’s cannabis industry as operator, producer, or policy stakeholder from June 2022 onwards; (b) direct experience with regulatory changes post-decriminalization; (c) Thai language proficiency; and (d) voluntary informed consent. Data collection continued until theoretical saturation was reached, in that later interviews did not generate new thematic content (Braun and Clarke 2006; Patton 2014) .

A total of 13 key informants were included in the study (7 females and 6 males; age range, 24–55 years; mean = 30.7 years), comprising 8 retailers, 3 cannabis farm operators, and 2 policy advocates. Characteristics of the study participants are given in Table 1.

**Table 1:** Participant characteristics (*n* = 13)

Code	Gender	Age	Role / Stakeholder Type	Operating Area
CB01	Female	26	Retail shop operator	Silom
CB02	Male	32	Retail shop operator	Sukhumvit
CB03	Female	25	Retail shop operator	Khao San Road
CB04	Male	55	Policy advocate	—
CB05	Male	27	Cannabis farm operator	BKK suburbs
CB06	Female	29	Retail shop operator	Sukhumvit
CB07	Male	28	Retail shop operator	Silom
CB08	Female	27	Retail shop operator	Khao San Road
CB09	Male	29	Retail shop operator	Sukhumvit
CB10	Female	24	Retail shop operator	Silom
CB11	Male	27	Cannabis farm operator	BKK suburbs
CB12	Female	28	Retail shop operator	Khao San Road
CB13	Female	42	Cannabis farm operator	BKK metro

*CB* Cannabis Business, *BKK* Bangkok

### Data collection

The key instrument of the research was a semi structured in-depth interview guide developed based on findings from a literature review of studies on cannabis policy, models for working with cannabis companies amid changing legislation, and the specifics of tourism related to cannabis consumption in Thailand. The following guide broadly includes five thematic areas: (a) experiences with laws and policy; (b) marketing/business strategies; (c) investment/risk management; (d) management/information access; and (e) sociology/culture. To enhance content validity, the guide was reviewed by three experts, including a cannabis policy researcher, an academic in health administration, and a practitioner in the cannabis industry. Some small refinements were made to the instrument after a pilot interview.

The principal investigator and a trained research assistant conducted in-depth interviews lasting 45–90 min each. Interviews occurred in settings selected by participants to facilitate confidentiality, typically at their place of work or at a mutually agreed-upon private location. Each session was audio-recorded and transcribed verbatim with prior written consent. Before analysis, the principal investigator checked each transcript for accuracy against the corresponding audio file.

### Data analysis

All data were analyzed using ATLAS. ti Version 21, using the six-phase framework for thematic analysis (TA) proposed by Braun and Clarke (2006).

During Phase 1 of data analysis, the PI read and re-read all transcripts while simultaneously creating analytic memos to document initial ideas.

In Phase 2, systematic open coding was applied across the dataset, with codes assigned to all semantically meaningful data units, enabling full coverage and detailed description.

Phase 3 involved carefully collating related codes into potential themes, providing a framework for further analysis.

Phase 4: These candidate themes were then refined against the entire dataset to ascertain their internal homogeneity and thematic uniqueness, making revisions as necessary to improve clarity and complexity.

Phase 5 involved collaboratively developing final theme names and definitions with the research team to ensure that each theme accurately reflected the underlying data.

Phase 6 resulted in a detailed analytic narrative that linked the findings back to what was known about the research objectives and provided a coherent account of how participants interpreted the data. A transparent, reproducible process was used, with an audit trail documenting all analytic decision-making. Disagreements between coders were resolved through consensus among the coding team.

### Trustworthiness

Trustworthiness was assessed against the four criteria outlined by Lincoln and Guba (1985). The data’s credibility was enhanced through member checking, which involved returning a summary of early findings to five participants for verification of the analytic deductions. Moreover, we applied data triangulation, by gathering input from operators of different types and locations. To enhance transferability, rich contextual descriptions were provided throughout the reporting process. Trustworthiness was established through an audit trail that meticulously recorded the research procedures. Confirmability was ensured through consistent researcher reflexivity; the lead investigator kept a reflexive diary documenting positional biases stemming from their public health policy background and strategies to minimize them.

## Results

This qualitative research applies observation and in-depth interview methods to gain reach information from key participants who operate retail shops in Bangkok’s business areas, namely Silom, Sukhumvit, and Khao San. Moreover, relevant stakeholders (e.g., farm operators, policy advocates, and supply-chain actors such as packaging manufacturers) were included to examine policy implications in the post-ban context. Interview data were managed and analyzed using ATLAS.ti.

### Overview of thematic framework

A thematic analysis of the in-depth interviews with 13 key informants revealed five primary themes characterizing the post-legalization cannabis landscape in Bangkok. These themes encompass the legal, economic, and socio-cultural dimensions of operating within a unpredictable regulatory environment.

#### Navigating regulatory unpredictability and legal uncertainty

The most salient theme identified was the tension between operators and an unpredictable regulatory landscape. Key Informants described a climate of “regulatory gaps” where legal uncertainties and shifting interpretations created substantial business risks. This unpredictability excessively burdened small-scale operators who lacked the capital to maintain compliance amidst frequent policy reversals. One participant (CB01) illustrated the operational tension of these changes:

*“The situation improved greatly after legalization… but then after they announced it was prohibited again*,* we had to post notices telling customers the law had changed—we were still operating but had to keep documenting everything.”* (CB01).

Despite these problems, operators emphasized that legalization improved institutional accountability, provided the state established a stable statutory framework.

*“Being legal is better for accountability*,* but there must be proper regulation—because everything here operates according to the law.”* (CB11).

The regulatory transition also created operational confusion at the point of sale. A Greenhead (Sukhumvit) retailer described having to inform customers directly about the change in status, a burden that fell on frontline staff rather than on any public information system:

*“Customers still come in*,* but we have to inform them that the law has changed—if they want to buy*,* they now need a patient certificate from a physician.” (CB02)*.

This placed operators in the position of communicating regulatory changes to the public in real time, in the absence of any formal government communication strategy directed at consumers.

#### Structural inequity and enforcement disparities

A significant sub-theme emerged regarding the “inequity of enforcement”. Participants—particularly local Thai operators—perceived a systemic bias toward large-scale corporations and foreign-invested entities. Specifically, the mandate for Good Agricultural and Collection Practice (GACP) standards was viewed as a barrier to entry designed for industrial pharmacy rather than local agriculture. As CB05 noted:

*“The law was designed to benefit pharmaceutical companies*,* requiring greenhouse farming standards… Thai farmers who grow and sell their own product must comply with GACP standards that were essentially designed for large operations.”* (CB05).

This perceived disparity extended to retail visibility, with one operator (CB13) highlighting that while foreign “chain stores” proliferated on Sukhumvit Road, small local shops faced more rigorous and frequent inspections.

*“Look at the equality issue—their shops are everywhere on Sukhumvit Road*,* 100% foreign brands—and yet we are left unchecked while small shops like ours face constant inspections.”* (CB13).

A staff member at a Silom dispensary similarly identified cost structures as a mechanism of exclusion, noting that the compliance requirements effectively created a two-tier market in which larger, better-resourced operators faced different standards than smaller shops:

*“A big shop has many things to pay for—employing a physician*,* staff costs*,* rent—but a small shop just gets a trading licence and starts selling; they are simply not held to the same standard.” (CB06)*.

This account aligns with the perspective of the policy advocate (CB04), who emphasised that the GACP greenhouse cultivation standard could only realistically be met by large pharmaceutical enterprises, leaving smallholder farmers and independent retailers at a structural disadvantage from the outset of the liberalised period.

#### Market segmentation and economic drivers

The legalization period triggered market growth, which participants largely attributed to Bangkok’s integration into global tourism flows. Analysis revealed two distinct customer segments.

- International Tourists: Comprising approximately 60% of the market, this group was characterized as experienced and knowledgeable users.
- Domestic Thai Customers: Comprising 40% of the market, these users were primarily motivated by medicinal or therapeutic needs.

*“We have both Thai and foreign customers—roughly 60% international and 40% Thai… Among users*,* there are two groups: those who have used before and those who want to try it.”* (CB13).

*“Most of our customers are foreigners who already use cannabis regularly*,* so they are quite knowledgeable.”* (CB08).

It should be noted that the 60/40 split cited by CB13 represents a practitioner estimate rather than verified commercial data, and was not uniformly shared across participants. Multiple operators at location-specific shops in Silom and Sukhumvit reported foreign customer proportions as high as 95–99%, reflecting differences in shop placement, pricing strategy, and tourist footfall by district. A Sukhumvit operator noted that Japanese tourists formed a particularly prominent sub-group within the medical-use segment:

*“Most of our Japanese customers come in because of insomnia or body aches from travelling or exercise—they are already familiar with cannabis from home but cannot use it legally there.” (CB09)*.

Peak trading periods were reported across multiple outlets, with daily revenues reaching 40,000–70,000 baht during Songkran and the year-end festival season—figures corroborated by several independent operators. Operators in the LGBTQ-adjacent entertainment district around Silom reported particularly high foot traffic, with one describing capacity constraints during major events.

Operators noted that peak trading cycles mirrored Bangkok’s established tourism seasons, specifically the end-of-year festival period.

#### Adaptation and survival strategies

Following the re-scheduling of cannabis and the restriction of promotional activities (such as “buy 3 get 1 free” deals), businesses were forced into rapid adaptation. Reported strategies included;

*“We had a promotion—buy 3 grams and get 1 gram free*,* along with member discounts.”* (CB09).

- Medical Integration: Shifting toward clinical models by collaborating with physicians to facilitate prescription-based access.
- Operational Contraction: Downsizing staff or permanent closure due to an inability to break even on heavy initial investments in facilities and quality control.

One operator (CB07) summarized the dire choice facing many: “The shop currently has two options: first, find a qualified person who can issue prescriptions; second, lay off most staff and operate alone”. Meanwhile another operator talked about the business’ struggling “In the first week, many shops temporarily closed, and some closed permanently because they could not find a physician to issue prescriptions.”

Operators who were affiliated with Thai traditional medicine networks demonstrated greater resilience through digital adaptation. One Sukhumvit operator described a telemedicine-linked dispensing model that allowed customers to obtain remote physician consultations and prescriptions through a QR-code system before purchasing:

*“Customers scan the QR code*,* an administrator takes their medical history*,* the physician consults them remotely*,* and then a prescription is issued—they can buy immediately after. We are part of a traditional medicine network so we can arrange this ourselves.” (CB02)*.

This kind of institutional alignment—combining retail cannabis operations with established medical provider networks—represented a significant competitive advantage that was unavailable to standalone or independent operators. Operators without such affiliations faced substantially higher transition costs and a narrower set of viable adaptation strategies.

#### Socio-cultural perceptions and public health

Cannabis remains a “socially sensitive” subject in Thailand due to decades of criminalization and resulting stigma. However, the data indicate a shift in the operators’ own attitudes, moving from viewing cannabis as a narcotic to framing it as a safer alternative to alcohol.

*“Cannabis is still a sensitive issue in Thai society—there is a great deal of stigma attached to it.”* (CB12).

*“I used to think of cannabis as a narcotic*,* but after working with it and trying it myself*,* I now believe that alcohol is more deserving of that classification.”* (CB10).

A consensus emerged regarding the urgent need for Youth Protection Measures. Operators advocated for a uniform statutory minimum age (e.g., 18–20 years) and mandatory ID verification to protect the industry’s legitimacy. Participant CB13 argued:

*“Large chain stores let minors purchase or offer no control whatsoever—if we are going to ban youth access*,* it must be enforced uniformly across all shops.”* (CB13).

A further sub-theme within Theme 5 concerns operators’ adoption of informal harm reduction practices. Contrary to the view that commercial promotion (such as purchase incentives during the liberalised period) is incompatible with harm reduction, the data suggest that both roles coexisted within the same operators at different points in the policy timeline. Promotional strategies were employed during the period of active liberalisation; harm reduction practices—including safety counselling, consumption space guidance, and management of adverse reactions—operated alongside and independently of commercial incentives. Operators at multiple sites described providing direct care when customers experienced adverse reactions from over-consumption:

*“The most common thing we see is tourists who have over-consumed and turn pale—they cannot move*,* or feel nauseated. We let them rest in the shop for two or three hours and make sure they are okay before they leave.” (CB03)*.

*“We always check the customer’s condition before we sell to them—if someone is already intoxicated when they come in*,* we will not sell to them*,* because if something happens*,* the shop is also responsible.” (CB02)*.

Operators also described proactively educating customers on safe consumption practices and local legal boundaries—for example, explaining that public smoking could result in fines of up to 25,000 baht or three months’ imprisonment. Staff at shops affiliated with the Thai Traditional Medicine Network reported structured induction training that included guidance on product effects, contraindications, and appropriate use by customer health profile. These practices were voluntary, operator-initiated, and without any formal public health mandate—underscoring the gap in institutional harm reduction infrastructure identified in the Discussion.

### Synthesis of evidence-based policy recommendations

Drawing directly from the thematic analysis and the lived experiences of Bangkok’s cannabis operators, six domains for evidence-based policy were synthesized. These recommendations serve as a practical framework for addressing the current regulatory and socio-economic challenges identified in the study.

#### Formalization of the regulatory and legal framework

Stakeholders advocated for the transition from transient decrees to a substantive Cannabis and Hemp Act to provide statutory clarity and consistent implementation.

- Age Controls: Establish a mandatory minimum age of 18 for purchase and sale, reinforced by enforceable verification requirements.
- Accessible Medical Schemes: Develop a prescription system that is operationally feasible and “convenient enough” for all parties to navigate.
- Standardized Enforcement: Ensure that quality and compliance standards are applied uniformly across all operators, regardless of their scale.

#### Promotion of regulatory equity

To counteract the perceived systemic bias favoring large-scale and foreign-invested entities, participants emphasized the need for market parity.

- Support for Small-Scale Operators: Implement tiered licensing fees and provide no-cost training on regulatory compliance to lower the barrier for local Thai businesses.
- Fair Market Oversight: Address the disparity where small shops face “constant inspections” while large international brands remain “unchecked”.

#### Enhancement of public health literacy

A structured information strategy is required to mitigate the historical stigma associated with cannabis and promote safe consumption.

- Educational Campaigns: Launch systematic, multi-channel public education efforts (both online and offline) to expand awareness of medicinal benefits.
- Professionalization: Support ongoing staff training through networks like the Thai Traditional Medicine Network (TTN) to ensure customers receive accurate, safety-centric guidance.

#### Robust youth protection mechanisms

The protection of minors emerged as a critical consensus among operators who expressed concern over current “no control” scenarios in large retail outlets.

- Verification Systems: Implement mandatory age checks at the point of sale using national identification systems.
- Proportionate Sanctions: Enforce strict penalties for operators found selling to minors to ensure accountability across the industry.

#### Product safety and quality standardization

Participants recommended clear, evidence-informed standards for processed products to ensure consumer safety.

- Edible Regulation: Establish regulated approval pathways for cannabis edibles (e.g., THC/CBD jellies) with clearly defined and controlled concentration thresholds.
- Practical Compliance: Ensure that quality standards are “achievable” for operators of varying sizes, rather than being tailored exclusively to industrial pharmaceutical models.

#### Sustainable cannabis tourism development

To leverage Thailand’s comparative advantages, stakeholders proposed a coherent tourism framework aligned with health and wellness.

- Health-Centric Identity: Cultivate a “responsible, health-centric destination identity” that differentiates Bangkok from international competitors.
- Traditional Medicine Integration: Align the sector with Thai Traditional Medicine and health-oriented services to attract high-value, specific interest groups.

## Discussion

This study provides qualitative evidence that Thailand’s cannabis policy transition did not merely alter the legal status of cannabis; it restructured the conditions under which cannabis-related tourism and retail could emerge, operate, and survive. Across participants’ accounts, the central issue was not legalization alone, but the instability of the regulatory environment in which legalization was introduced and then partially reversed. In Bangkok, this instability shaped market entry, business adaptation, perceptions of risk, and the unequal distribution of opportunity across operators. The findings therefore suggest that the Thai case is best understood not simply as a story of cannabis liberalization, but as a case of institutionally unstable market formation in which legal ambiguity itself became a decisive force shaping commercial viability, enforcement exposure, and public health practice. This pattern is consistent with broader evidence showing that cannabis regulation remains difficult to implement coherently across jurisdictions, especially where access, prescribing, product control, and oversight mechanisms evolve unevenly over time (de Souza et al. 2022).

The findings also demonstrate the analytical value of using Butler’s Tourism Area Life Cycle (TALC) together with the concept of the policyscape. Viewed through TALC, Bangkok’s cannabis tourism economy exhibited a compressed and disrupted development trajectory: rapid expansion following decriminalization was followed not by gradual consolidation, but by abrupt policy-induced stagnation. This extends Butler’s framework by showing that destination trajectories may be truncated not only by market saturation or environmental decline, but also by politically driven regulatory reversal. At the same time, the policyscape lens helps explain why operators experienced the legal environment as fragmented, burdensome, and difficult to navigate. Rather than responding to a single coherent regime, participants described managing a layered institutional terrain in which older medical cannabis rules, newer decriminalization measures, and shifting enforcement expectations coexisted without effective integration. Recent re-engagement with the TALC model also supports its continued relevance for analyzing destination change, while leaving room for adaptation to contemporary disruptions not fully addressed in the original model, including sudden exogenous shocks and governance instability (Butler 2025). Taken together, these frameworks illuminate both the temporal disruption of cannabis tourism development and the unequal institutional burdens imposed on different categories of operators.

A major contribution of this study is its demonstration that regulatory ambiguity functioned as a structural mechanism of inequality. Participants consistently described the absence of a comprehensive Cannabis and Hemp Act as producing uncertainty around compliance, investment planning, and day-to-day operations. However, these burdens were not experienced evenly. Smaller Thai operators were more likely to report difficulties interpreting new requirements, absorbing compliance costs, and sustaining operations under rapidly changing conditions, whereas larger or foreign-backed enterprises were perceived as better able to withstand institutional complexity. In this sense, legal ambiguity did not simply create confusion; it redistributed market advantage toward actors with greater capital, legal knowledge, and administrative capacity. This finding shifts the discussion from regulatory “uncertainty” as a neutral condition to regulatory instability as a process that can actively intensify inequality within an emerging cannabis sector.

The financial consequences described by participants further reinforce this interpretation. Many operators had invested substantially in cultivation systems, retail infrastructure, staffing, and product development during the liberalized period, only to face a sharp change in the rules governing sales and market access. The need to pivot quickly toward medical-oriented business models, secure prescription-linked access, reduce staffing, or close altogether indicates that policy reversal generated not only compliance challenges but sunk-cost losses. Importantly, participants’ accounts suggest that these losses were especially damaging for less-capitalized businesses that lacked diversified revenue streams or institutional buffers. The findings therefore support the argument that transition frameworks in cannabis governance should be evaluated not only in terms of legality and public health intent, but also in terms of how predictably they allow businesses to adapt without disproportionate harm to smaller domestic actors.

Another important finding is that cannabis retail in Bangkok was deeply entangled with tourism. Participants described a customer base heavily reliant on foreign visitors, peak sales during festival and high-travel periods, and rapid commercial expansion in areas already central to Bangkok’s tourism economy. These accounts suggest that cannabis retail became incorporated into the city’s urban tourism infrastructure with notable speed. Yet this integration also made the sector highly vulnerable. Because demand was closely tied to international mobility and tourist consumption, regulatory tightening had consequences that extended beyond cannabis policy itself and into the wider organization of tourism-linked commerce. This tourism entanglement is consistent with emerging cannabis tourism scholarship showing that legalization can become part of destination image formation, tourist motivation, and broader debates over how to balance economic opportunity with health and social responsibility (Dupej and Choi 2025; Liang et al. 2023). This is important conceptually because it shows that cannabis tourism in Bangkok was not a marginal or isolated niche; it was embedded in mainstream urban tourism circuits, but without the stable governance architecture usually required for sustainable destination development.

The study further indicates that operators were not merely passive recipients of regulatory change. They actively adapted their business models, marketing practices, and customer engagement strategies in response to the evolving policy environment. Promotions, loyalty schemes, and tourist-oriented sales practices were reportedly common during the liberalized phase, then abruptly suspended once enforcement tightened. This rapid recalibration highlights the sensitivity of emerging cannabis markets to state signals, especially where legal and reputational risks remain high. More broadly, it suggests that business adaptation in transitional cannabis economies is shaped less by long-term strategic planning than by short-cycle responses to regulatory volatility. That dynamic is particularly consequential in tourism-linked markets, where consumer demand may remain strong even as permissible commercial practices become more restricted or ambiguous.

One of the study’s most original findings is the emergence of operators as informal harm reduction actors. This role is consistent with evidence from legal retail settings showing that dispensary staff often understand their work as involving not only sales, but also customer education, product guidance, and communication about safer use and legal compliance (Carlini et al. 2022). Participants described providing staff training, explaining product effects, updating customers on regulatory changes, and guiding inexperienced users to reduce the likelihood of adverse experiences. In the absence of fully institutionalized retail education standards or widely accessible public health communication, these practices appear to have functioned as practical substitutes for formal harm reduction infrastructure. This is a notable contribution because cannabis retailers are often discussed primarily as commercial actors, whereas the present findings show that, in transitional settings, they may also assume quasi-public-health roles. Similar findings have been reported in other legal cannabis contexts, where retailers describe licensing, pricing, and distribution rules as materially shaping business sustainability and the extent to which smaller actors can participate on equitable terms (Wright-Brown et al. 2025). That does not mean such functions should be left to businesses alone. Rather, the data suggest that future regulation should acknowledge this reality and incorporate standardized training, clearer safety communication requirements, and government-led educational support so that harm reduction does not depend on the variable discretion or capacity of individual operators. More broadly, the findings align with public-health scholarship showing that legalization can expand access and commercial opportunity while also increasing the importance of retail regulation, youth protection, product standards, and public education (Hall and Lynskey 2020).

Concerns about youth access further underscore the need for a more coherent regulatory architecture. Participants repeatedly emphasized age verification, identification checks, and consistent enforcement, but they also perceived substantial unevenness in how youth protection rules were applied across shops. Particularly striking was the view that smaller operators were scrutinized more closely while larger chain-style businesses were able to evade equivalent oversight. Whether or not such perceptions fully reflect enforcement practice, they are analytically important because they reveal how legitimacy is experienced at street level. For participants, youth protection was not framed as a reason to oppose regulation; rather, it was presented as a reason to demand clearer and fairer regulation. These concerns are aligned with emerging Thai evidence showing increases in cannabis use following policy liberalization and associations between outlet density and current cannabis use, as well as broader review evidence linking greater physical availability of retailers with more frequent use and related harms (Cantor et al. 2024; Kalayasiri and Boonthae 2023; Wichaidit et al. 2024). This distinction matters. It suggests that operators were not rejecting oversight per se, but contesting a system in which enforcement appeared selective, inconsistently implemented, and insufficiently aligned with the stated goals of public protection.

The findings also highlight the persistence of stigma despite legal reform. Participants described cannabis as remaining socially sensitive in Thailand, with older narcotics-based understandings continuing to shape public perceptions and constrain broader acceptance. At the same time, several operators reported substantial personal shifts in how they understood cannabis, moving from seeing it primarily as a dangerous drug to recognizing medicinal or wellness-related applications. This tension between legal change and cultural lag is significant. It suggests that policy change alone does not automatically transform the symbolic status of cannabis; instead, social meanings are renegotiated unevenly across institutions, communities, and everyday interactions. This is consistent with growing evidence that both external stigma and self-stigma may persist even after legal reform, and that stigma continues to shape disclosure, legitimacy, and help-seeking around cannabis use (King et al. 2024; Rosenkranz et al. 2025). In the Thai case, this cultural transition appears to be shaped not only by law, but also by media narratives, tourism imaginaries, and the extent to which the state provides credible public information. The implication is that successful cannabis governance requires not only legal clarity, but also sustained communication strategies that address stigma, misinformation, and divergent public understandings of legitimate use.

More broadly, Bangkok’s case offers insights that extend beyond Thailand. Much of the existing cannabis literature has been developed in North American or European settings, where regulatory pathways, institutional capacity, and market structures differ substantially from those in Southeast Asia. This study shows that in an emerging Asian cannabis market closely linked to tourism, the key governance challenge may not be legalization versus prohibition as such, but the sequencing, coherence, and enforceability of reform. The Bangkok case is especially instructive because rapid market growth occurred before a stable regulatory settlement was in place, creating a situation in which commercial expansion, public health concerns, and political contestation unfolded simultaneously. This makes the case useful not only for understanding Thailand, but also for informing other jurisdictions considering reform under similarly contested institutional conditions.

Taken together, the findings suggest that future cannabis policy in Thailand should move beyond reactive control measures toward a more integrated governance model. Such a model would require, at minimum, clear statutory rules, proportionate and equitable enforcement, feasible compliance pathways for smaller operators, standardized product and retail safety requirements, and public education that addresses both health literacy and stigma. Without these elements, regulatory instability is likely to continue reproducing the same problems identified by participants: unequal enforcement, commercial vulnerability, patchwork harm reduction, and uncertainty about the legitimate place of cannabis within Thailand’s tourism and health landscape. In that sense, the most important lesson from this study is that the sustainability of cannabis tourism depends not simply on whether cannabis is permitted, but on whether the policy environment is coherent enough to support fair, safe, and socially legitimate implementation.

Finally, the rapid and largely unfettered commercialization of cannabis in Bangkok has brought the critical issue of high-potency cannabis to the forefront of national public health and policy debates. While the 2022 decriminalization framework intended to restrict high-potency products by enforcing a 0.2% THC cap on cannabis *extracts*, this threshold completely omitted raw cannabis inflorescences (buds). Consequently, unextracted plant material from high-potency strains has been legally and widely retailed without any statutory potency limits (Yimsaard et al. 2023). Because there is currently no systematic monitoring, mandatory laboratory testing, or standardized labeling of THC strength within retail settings, the exact potency of products available to consumers remains a critical blind spot. This absence of retail-level oversight represents a notable and hazardous gap in public health governance, serving as a direct consequence of the overall lack of a comprehensive regulatory framework during the initial decriminalization phase. Without centralized testing mandates, both compliant operators and consumers are left to navigate an unregulated market with unverified products. This structural deficit underscores the urgent need for a formalized, state-enforced regulatory system that incorporates mandatory quality control and potency testing to mitigate public health risks—particularly regarding vulnerable youth populations—while establishing a predictable, standardized market for industry stakeholders.”

### Limitations

Several limitations of the present study merit acknowledgement. First, the sample size of 13 key informants, while consistent with established conventions for in-depth qualitative inquiry and sufficient to achieve theoretical saturation, necessarily limits the breadth of perspectives represented. Cannabis business operators in Bangkok are a heterogeneous population, and certain sub-groups—including undocumented or informal operators, foreign-national owners, and operators who exited the market prior to the fieldwork period—are unlikely to have been reachable through the purposive and snowball sampling strategies employed. The sample may therefore over-represent operators who remained in the market and were willing to discuss their experiences, potentially introducing survivorship bias into the findings.

Second, the geographical scope of data collection was limited to Bangkok, which, while the primary site of cannabis retail activity in Thailand, does not capture the experiences of operators in other cannabis tourism destinations such as Pattaya, Chiang Mai, Phuket, or Samui—markets that may exhibit distinct structural characteristics, customer demographics, and enforcement dynamics. Findings should therefore be interpreted as specific to Bangkok’s urban cannabis tourism context and may not transfer directly to other Thai jurisdictions.

Third, data collection coincided with a period of acute regulatory uncertainty (2024–2025), during which some participants may have experienced heightened caution or social desirability bias in disclosing the details of their operations, given that the legal status of recreational cannabis remained contested throughout the fieldwork period. Snowball sampling, while useful for accessing a hard-to-reach population, may also have introduced network homophily, whereby referrals clustered within segments of the industry—for example, among operators sharing regulatory concerns or business models.

Future research should consider longitudinal designs to compare outcomes for operators who adapted to re-scheduling against those who exited the market, and to assess the public health impacts of different retail cannabis education models over time. Comparative studies across Southeast Asian jurisdictions—including evolving reform discussions in Malaysia, Indonesia, and the Philippines—would further strengthen the regional evidence base for cannabis policy design. Health equity dimensions of Bangkok’s cannabis industry also warrant dedicated investigation, given the structural inequities in enforcement, investment capacity, and market access documented in this study.

## Conclusion

This qualitative study examined the experiences of cannabis business operators in Bangkok during Thailand’s transition from cannabis decriminalization to a medical-only regulatory regime. Thematic analysis of 13 in-depth interviews identified five primary themes spanning legal, economic, and sociocultural dimensions of this policy transition. Three findings carry particular policy significance. First, the absence of a comprehensive Cannabis and Hemp Act created persistent legal ambiguity that imposed disproportionate compliance burdens on small Thai operators, exacerbating structural inequalities relative to foreign-backed enterprises. Second, the tourism-dependent nature of Bangkok’s cannabis retail sector rendered businesses acutely vulnerable to both regulatory instability and shifts in international travel demand. Third, in the absence of formal public health infrastructure, operators assumed informal harm reduction roles through staff training and proactive customer communication—functions that warrant explicit recognition in future regulatory design. These findings contribute original qualitative evidence on cannabis tourism governance in Asia, where comparable scholarship remains scarce. Bangkok’s experience as a first-mover jurisdiction offers transferable lessons for other countries navigating cannabis policy reform. The enactment of clear, equitable, and practically implementable cannabis legislation remains the most critical precondition for sustainable development of this sector.

## Data Availability

The original contributions presented in the study are included in the article and the data that support the findings of this study are available. However, regarding to ethical and privacy considerations, access to certain portions of the data is restricted. For inquiries regarding data access, please contact the corresponding author, Dr Pattarachit Choompol Gozzoli, email pattarachit.goz@mahidol.edu.
